# Nanopipette-assisted single cell metabolic glycan labeling†

**DOI:** 10.1039/c9ra06634a

**Published:** 2019-09-27

**Authors:** Ze-Rui Zhou, Xiao-Yuan Wang, Jian Lv, Bin-Bin Chen, Yi-Bin Tang, Ruo-Can Qian

**Affiliations:** Key Laboratory for Advanced Materials, School of Chemistry & Molecular Engineering, East China University of Science and Technology Shanghai 200237 P. R. China ruocanqian@ecust.edu.cn +86 21 64250032

## Abstract

Here, we report a single cell glycan labeling strategy by combining nanoscale intracellular glass electrodes with bioorthogonal reaction. With the tip diameter less than 100 nm, the nanopipette electrode can be spatially controlled to inject artificial monosaccharides into single living cells with minimal invasion. The injection process can be precisely regulated by electroosmotic flow inside the nanopipette, and fluorescence labeling of sialic acid at single cell level is achieved.

## Abstract 2

This work reports a single cell glycan labeling strategy based on nanopipettes, which provides a convenient, fast labeling method for bridging the gap between single cell manipulation and metabolic labeling.

## Graphical abstract

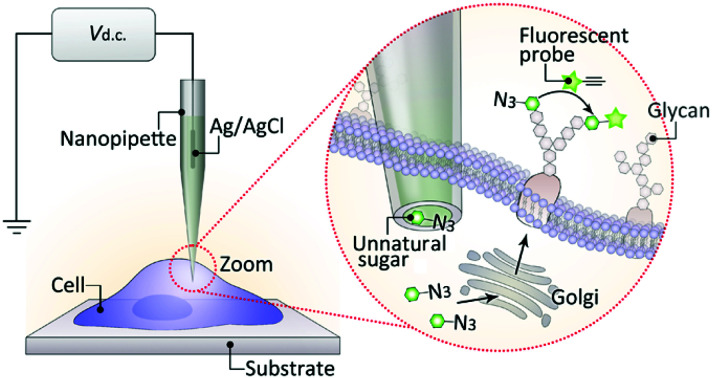

The surfaces of all cells are decorated with a dense array of sugar chains.^1^ Cell surface glycans play important roles in regulating a variety of biological functions, such as structure composing, cell–cell recognition, and disease development.^2^ As the precursors for various glycoconjugates, monosaccharides are ingested by cells for the construction of diverse glycan structures.^3^ Therefore, probing the expression of glycans on cell surfaces can provide valuable information for the elucidation of their functional roles and impact on human diseases. Key strategies for glycan labeling have emerged by using the lectins extracted from plants or animals through lectin-saccharide recognition.^4^ Recently, a thriving community of researchers use metabolic labeling for the detection of cell surface glycans, which greatly improves the specificity and imaging effect.^5^ Nevertheless, exogenous sugars used in metabolic labeling usually enter cells without discrimination, resulting in global labeling of glycans in a large number of cells. Besides, it is time consuming to complete the whole process of metabolic labeling, which takes more than 24 h. These limitations negate the ability of the traditional metabolic labeling method to study glycan expression profiles at single cell level.

It is crucial to study the glycan expression diversity of seemingly identical cells for enhancing our understanding of the genetic heterogeneity at single cell level.^6^ Currently, significant advances have been obtained in the development of single cell techniques, especially the strategies for single cell imaging.^7^ Nanoscale devices such as nanowires, nanotubes and nanoelectrodes have been developed and have shown great power for single cell research due to their high spatial and temporal resolution.^8^ Notably, the insertion of non-destructive devices based on glass nanopipettes enables the analysis of specific molecules in single living cells.^9^ Nanopipettes are convenient to fabricate and easy to use. With the ultra-small tip areas (diameter less than 100 nm), nanopipettes provide a favorable tool for the precise location and transportation targeting single living cells with little damage.^10^

In this work, we report the development of a single cell targeted metabolic glycan labeling strategy based on the glass nanopipettes with minimally invasion (Fig. 1a). An Ag/AgCl electrode was inserted into the nanopipette as the working electrode, and another Ag/AgCl was immersed in the culture medium as the reference electrode. The ultra-small tip of the nanopipette enabled the precise localization of a selected single living cell. The nanopipettes utilized electroosmotic flow to inject artificial saccharide molecules into the cytoplasm. The electroosmotic flow of the sugar solution inside the nanopipette can reach ∼11 fL s^−1^ under 400 mV positive DC voltage, which enabled the ultra-fast influx of exogenous monosaccharide in less than 5 min, thus greatly shortened the transferring time (usually more than 24 hours using traditional metabolic labeling) to ∼6 h. Our strategy has expanded the utility of nanopipettes to single cell glycan labeling and manipulation. The validity of the strategy was confirmed by performing the injection of artificial azide-tagged mannose (ManNAz, expressing SiaNAz on cell surface through the incorporation into the sialic acid biosynthetic pathway)^3*a*,5*a*^ and the subsequent bioorthogonal fluorophore marking *via* the azide/alkyne click reaction. Therefore, the nanopipette based metabolic labeling may open a way for the efficient manipulation of glycans at single cell level, thus promoting the study of glycobiology and the related cell pathways.

**Fig. 1:**
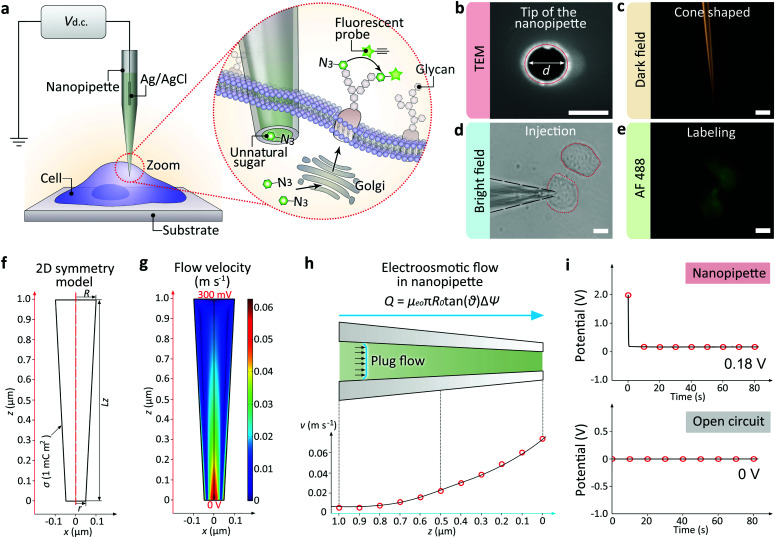
(a) Illustration of the single cell glycan labeling by a nanopipette. Application of a positive DC voltage enables the ultra-fast injection of exogenous azide-tagged monosaccharides, which can be marked by fluorophore *via* the azide/alkyne click reaction. (b) Scanning electron microscope (SEM) image showing the tip hole of the nanopipette. Scale bar, 100 nm. (c) Dark field (DF) microscopic image showing the cone-shaped tip of the nanopipette. Scale bar, 10 μm. (d) BF image showing a cell inserted by the nanopipette. Scale bar, 20 μm. (e) Fluorescent microscopic image showing an alkyne-AF488 (green) labeled cell. Scale bar, 20 μm. (f) 2D symmetry model of the nanopipette. (g) 2D velocity field inside the nanopipette. (h) Plug-shaped electroosmotic flow (up) and the velocity alone the *z*-axis. (i) The nanopipette and the Ag/AgCl electrode in the culture medium (up) or the open circuit potential between two Ag/AgCl electrodes (down).

The nanopipettes we used here were obtained by pulling clean quartz capillaries (Fig. S1†). As shown in the scanning electron microscope (SEM) image, the tip hole was around 100 nm (Fig. 1b). With this tip size, the liquid inside the nanopipette can enter into the cell smoothly while maintaining cell morphology (Fig. S2 and S3†). The cone-shaped tip of the nanopipette could be observed clearly under the dark field microscope (Fig. 1c).^11^ For single cell injection, the nanopipette was filled with azide-tagged artificial saccharide solution, then approaching the cell surface slowly under the control of a micromanipulator (Fig. S4†). After the tip entered into the selected cell (Fig. 1d), the application of a DC voltage generated an electric field gradient (Fig. S5†), which created an electroosmotic-driven outflow near the tip for the effective injection and following metabolic labeling (Fig. 1e). From finite element method calculations based on a 2D symmetry model of the cone-shaped nanopipette (Fig. 1f), flow velocity as high as 0.07 m s^−1^ at the tip could be obtained (Fig. 1g) under 400 mV, with the liquid flux reaching 11 fL s^−1^ (Fig. 1h). The open circuit potential of the nanopipette was −200 mV (Fig. 1i).

The effectiveness of the nanopipettes for metabolic labeling in single cells was firstly confirmed using MCF-7 breast cancer cells. Azide-tagged ManNAz was injected into a selected cell for the expression of SiaNAz on cell surface and the following click fluorescence labeling using alkyne-AF488 (Fig. 2a). The expression time of glycans could be reduced to less than 6 h (Fig. 2b), and the operation time for the whole labeling process was significantly shortened to less than 6.5 h (30 min for click fluorescence labeling) compared with the traditional metabolic labeling (more than 24 h, Fig. S6†). Next, different injection times was studied under positive 400 mV DC voltage. As shown in Fig. 2c, 5 min was enough to inject sufficient ManNAz for the SiaNAz expression. The application of a series of different voltages from 200 mV to 800 mV showed an increased SiaNAz expression with the growing voltage, until a maximum was reached at 600 mV (Fig. 2d). As a control, no fluorescence could be seen without applying any voltage (Fig. 2e), which confirmed the electroosmotic-driven outflow. Statistics of the average fluorescence intensity within a single cell was shown in Fig. 2f. As expected, the labeling efficiency was dependent on the expression time, injection time and the applied voltage. Combining the above aspects, the optimized conditions for single cell glycan labeling was as following: 5 min injection, 400 mV positive voltage, 6 h expression and 30 min click fluorescence marking (Fig. 2g). Matlab r2017b was used for acquiring 2D and 3D color-coded pseudograms (Fig. S7†). A typical 3D fluorescence distribution of a single MCF-7 cell under the optimized condition was shown in Fig. S8.†

**Fig. 2:**
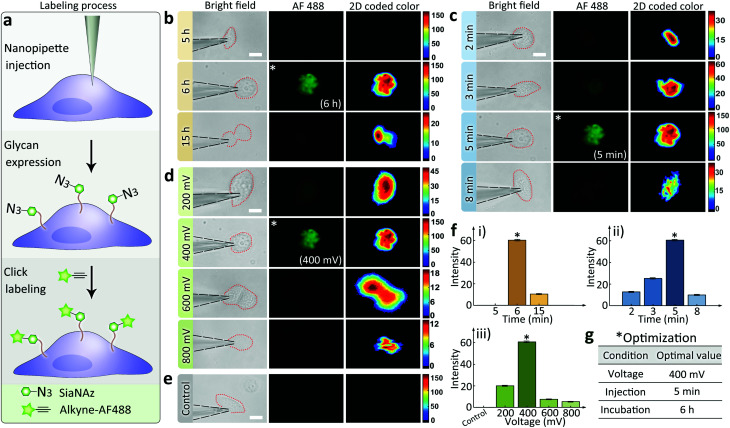
(a) Schematic of the monosaccharide expression process. Following nanopipette injection of an artificial monosaccharide ManNAz, SiaNAz was expressed on the surface of a selected cell, and then tagged by alkyne-AF488 (green). Microscopic images showing SiaNAz level under (b) various expression times (from up to down: 5, 6, 15 h); (c) various injection times (from up to down: 2, 3, 5, 8 min); (d) various injection voltages (from up to down: 200, 400, 600, 800 mV) & (e) control. Column from left to right (b–e): Bright field, green fluorescence, 2D color-coded green channel intensity. Scale bar, 20 μm. (f) Average green channel intensity of MCF-7 cells corresponding to different conditions in (b), (c) and (d) ((i): (b), (ii): (c), (iii): (d)). *Optimized condition (b–e). (g) Optimized conditions for the nanopipette labeling experiments.

Having demonstrated the ability of labeling glycans on single cell surface *via* nanopipettes, next we studied whether the glycan expression pathway including endocytosis and the subsequent membrane expression could be influenced by the cell type. To do this, we simultaneously studied three types of cells, including MCF-7 breast cancer cells, HeLa cells, and RAW264.7 macrophage cells (Fig. 3). As a control, we first studied the final SiaNAz expression using traditional metabolic labeling, as the expression time was 24 h. After labeling *via* alkyne-AF488, the fluorescence images were observed. The average fluorescence intensity within the cell area was obtained and then corrected by multiplying a calibration factor (the third power of relative diameter for volume correction). As shown in Fig. 3a–c, expression level of SiaNAz was highest in HeLa cells, lower in MCF-7 cells, and lowest in RAW264.7 cells, which confirmed higher SA expression in cancer cells. Next, we studied the nanopipette based single cell labeling. In contrast, the fluorescence signal intensities of these cells were much higher than those marked by traditional metabolic labeling, and the average SA expression level was quite similar in three cell lines (Fig. 3d–f). From the above results, we can see that the nanopipette based labeling is more efficient than traditional method. In addition, since nanopipette injection can transport the same amount of ManNAz in each cell under the same experimental parameters, we speculate that once the ManNAz molecules enter into the cytoplasm, they will be transferred to SiaNAz and expressed on cell surface *via* the sialic acid biochemical pathway.^12^ In contrast, if the ManNAz molecules are dispersed in the culture solution, the SiaNAz expression is different in various cell lines. These findings thus confirmed the rapid and convenient labeling of glycans based on nanopipettes in different types of cells.

**Fig. 3:**
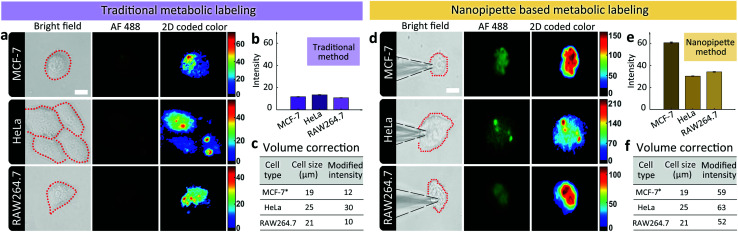
(a) Microscopic images showing the expression levels of SiaNAz in different cell lines using traditional metabolic labeling method (from up to down: BF images of cells after incubating with ManNAz for 24 h, fluorescence images of green channel, 2D color-coded green channel intensity, 3D green channel intensity). Scale bar, 20 μm. (b) Average green channel intensity of different cells in (a). (c), Corrected average SA expression in different cell lines. (d) Microscopic images showing the expression levels of SiaNAz in different cell lines using nanopipette labeling method (from up to down: BF images of cells after injection for 6 h, fluorescence images of green channel, 2D color-coded green channel intensity, 3D green channel intensity). Scale bar, 20 μm. (e) Average green channel intensity of different cells in (d). (f) Corrected average SA expression in different cell lines. *MCF-7 cell was selected as the standard for correction by setting the diameter of MCF-7 as the basic unit with value of 1. Thus the relative diameter of HeLa was 1.3 and RAW264.7 was 1.1. The average fluorescence intensity with the cell area was corrected by multiplying a calibration factor (the third power of relative diameter for volume correction).

Next we returned to the labeling of SiaNAz using nanopipettes in MCF-7 cells. After successful labeling under the optimized conditions, the injected cells were further observed for more than 6 h. Interestingly, we found that an asymmetric cell division occurred, as two daughter cells had different cellular fates. As shown in Fig. 4, in contrast to symmetric cell divisions generating two daughter cells of equivalent cell fates, the injected MCF-7 cell tended to divide in less than 6 h, and generated a new daughter cell alongside. After another 3 h, the original injected MCF-7 cell divided again to give one more cell of the third generation, while the other daughter cell did not divide (Fig. S9†). As it has been reported that cancers contain rare subpopulation of cancer stem cells, which divide asymmetrically,^13^ the asymmetric division in MCF-7 cells might be attributed to the overexpressed SiaNAz after artificial injection. In this case, we speculate that the higher level of cell membrane SiaNAz made the MCF-7 cell easier to divide, and may converting these cells into cancer stem cell-like cells.

**Fig. 4:**
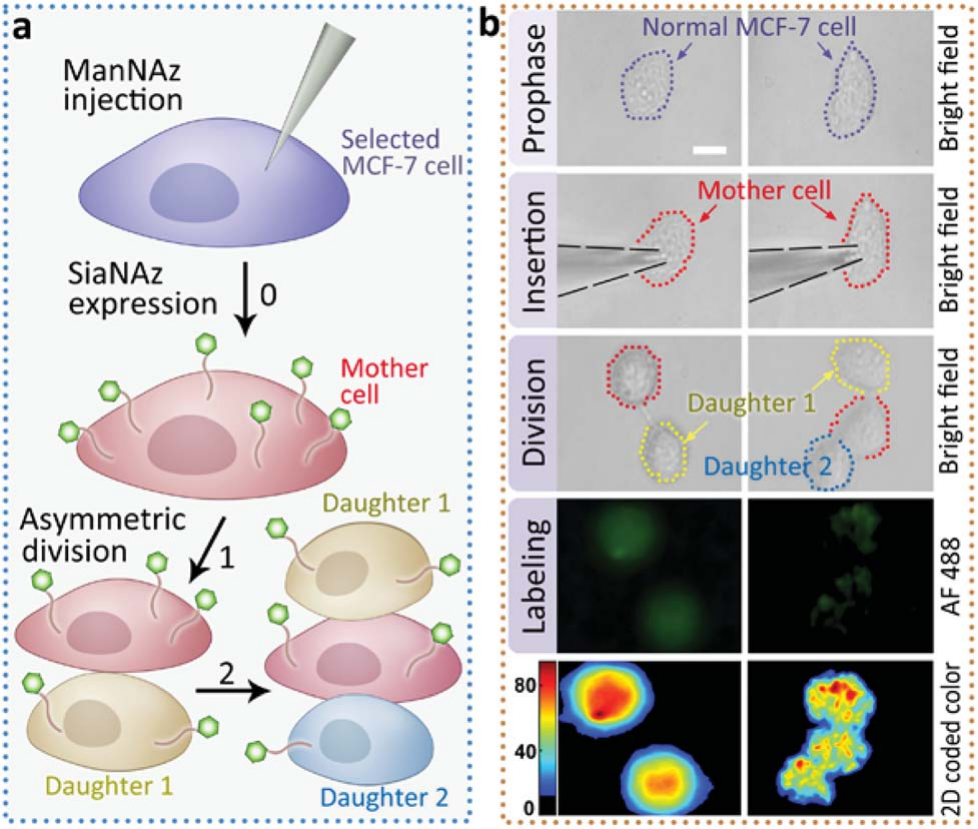
Asymmetric division of ManNAz injected MCF-7 cells. (a) Schematic of the asymmetric division in the ManNAz injected MCF-7 cell. A selected MCF-7 cell was injected by a nanopipette containing ManNAz for the expression of SiaNAz, and then tagged by alkyne-AF488 (green). Asymmetric cell division occurred. (b) Microscopic images showing the process of asymmetric division and the corresponding SiaNAz level (from up to down: BF images of a selected MCF-7 cell before injection, during injection, the cell under division, green fluorescence, 2D color-coded green channel intensity). Scale bar, 20 μm.

In conclusion, we have demonstrated the fabrication and application of nanopipettes for glycan labeling and engineering on single cell surface in physiological environments. The nanopipettes are cheap and convenient to prepare. The ultra-small tip enables the precise pointing of a single living cell. Using a DC voltage, electroosmotic-driven flow can be generated for the fast injection of artificial glycans into a single cell in less than 5 min, which significantly reduced the transferring time of exogenous sugars. Further, we compared our injection strategy with the traditional metabolic labeling, and demonstrated the validity and commonality of the nanopipette based method in different cell lines. Additionally, we also observed the asymmetric division of MCF-7 cells after ManNAz injection, which made the injected cells obtaining features of cancer stem cells. The nanopipette based technology allows us to manipulate glycan expression at single cell level, which would be further used for the research of glycobiology and glycan related cell behavior.

## Conflicts of interest

There are no conflicts to declare.

## Supplementary Material

RA-009-C9RA06634A-s001
