# Supplementary material for: Nanopipette-assisted single cell metabolic glycan labeling
Source: RSC Adv. 2019 Sep 27;9(53):30666–70. doi: 10.1039/c9ra06634a (PMC9072180; doi:10.1039/c9ra06634a)
Supplement: RA-009-C9RA06634A-s001 [file RA-009-C9RA06634A-s001.pdf]

Electronic Supplementary Material (ESI) for RSC Advances.
This journal is © The Royal Society of Chemistry 2019

Electronic Supplementary Information

Nanopipette-Assisted Single Cell Metabolic Glycan Labeling

Ze-Rui Zhou, Xiao-Yuan Wang, Jian Lv, Bin-Bin Chen, Yi-Bin Tang and Ruo-Can Qian*

East China University of Science and Technology

*E-mail: ruocanqian@ecust.edu.cn

Supplementary Methods

Materials and Reagents

All the materials and reagents are of analytical grade, and solvents were purified by standard procedures. All solutions were prepared by Milli-Q ultrapure water with resistance of 18.2 M Ω cm at 25 ℃ (EMD Millipore, TONDINO, Shanghai). Click-iT® tetraacetylated N-azidoacetyl-D-mannosamine (ManNAz), and Alexa Fluor® 488 alkyne (AF 488-alkyne) were purchased from Thermo Fisher (USA). Normal 1640 cell media, DMEM cell media, 10% Fetal Bovine Serum and antibiotics were purchased from MoXi Biotech. Co. Ltd. (Shanghai, China). MCF-7, HeLa and Raw-267 cell lines were purchased from cells were from MoXi Biotech. Co. Ltd. (Shanghai, China). Phosphate buffer saline (PBS, pH 7.4) contained Dibasic Sodium Phosphate and Sodium Dihydrogen Phosphate were purchased from Aladdin. Quartz capillaries (O.D.: 1.0 mm, I.D.: 0.7 mm; 7.5 cm length) were purchased from Sutter Instrument. The quartz capillaries were cleaned first by pure water, and then washed successively in ethanol and pure water with at least 40 min sonication. Lastly they were dried by nitrogen gas. The nanopipettes were backfilled by using a microloader (Eppendorf). Blue tacks were purchased from the Chinese agency of Photo Tack.

Apparatus

A P-2000 laser puller (Sutter Instrument, Novato, CA) was used for fabricating nanopipettes in the experiment. Optical bright-field spectrum measurements were used to record by using a Nikoneclipse Ti-U inverted microscope equipped with a 40 × objective lens (NA = 0.8), which was used to obtain the spectra in a technological channel. A true-color digital camera (Nikon, DS-fi, Japan) was used for recording the field of the microscope for coregistration with the monochromator. The holder (Axon Instruments, Union City, CA) was used to fix the nanopipette. The holder was connected to an Axopatch 700B low-noise amplifier (Molecular Devices, Sunnyvale, CA) for current measurement, with a MP-285 micromanipulator (Sutter Instrument, Novato, CA) for coarse control of the nanopipette positioning in the X, Y, and Z directions, a Nanocube piezo actuator (Physik Instrument, Irvine, CA) for fine control in the X, Y, and Z directions, and a PCIe-7851R Field Programmable Gate Array (FPGA) (National Instruments) for hardware control of the system. The whole system was operated using custom coded software written by LabVIEW.

Cell culture

Hela cells were cultured in DMEM (GIBCO) with 10% fetal bovine serum (FBS, Sigma) and antibiotics. MCF-7 and RAW264.7 cells were cultured by RPMI-1640 (GIBCO) with 10% fetal bovine serum (FBS, Sigma) and antibiotics. The culture dishes were placed in a humid atmosphere at 37 ^{o}C with 5 % CO_2.

Supplementary Note 1. Fabrication of Nanopipettes

The nanopipettes described herein were fabricated from quartz capillaries (with outside diameter of 1.00 mm and inside diameter of 0.70 mm (Sutter Instrument, Novato, CA) by using a P-2000 laser puller (Sutter Instrument, Novato, CA) according to relevant references.[1] The pulling parameters were regulated by a custom two-line program (Line1: heat-650, fil-3, vel-35, del-145 and pul-75; Line2: heat-920, fil-2, vel-15, del-128 and pul-200). The mean diameter of the nanopipette tip pore was about 100 nm. It should be pointed out that the pulling parameters are instrument dependent and may be different from puller to puller.[2]

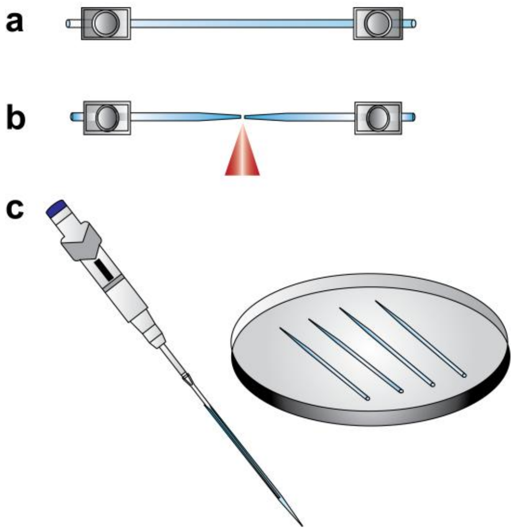

Figure S1. Illustration showing the nanopipettes set-up. (a) The quartz capillaries were fixed by a horizontal holder. (b) A laser beam was used for heating the middle part of quartz capillaries, and then the tubes were stretched on both sides for the formation of two conical nanopipettes. (c) The obtained nanopipettes were fixed on a holder for the cell inserting experiments. Nanopipettes were kept in a clean culture dish.

Supplementary Note 2. Selection of the Nanopipette Tip Diameter

In our experiments, we need to inject electroneutral sugars into single living cells. If the tip diameter is too small (< 100 nm), the tip hole is likely to be blocked during the injecting process (Fig. S2). Since the cell damage caused by the 100 nm nanopipette is negligible (Fig. S3), we use 100 nm nanopipettes in our experiments to ensure that the liquid can enter into the cell smoothly while maintaining cell morphology.

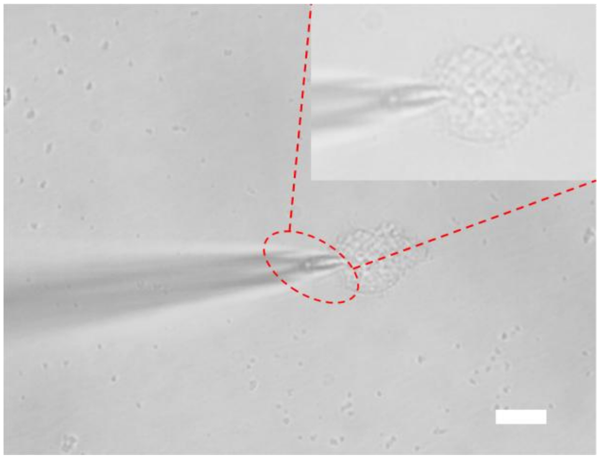

Figure S2. Blockage of the 50 nm nanopipette tip when injecting a living MCF-7 cell. Scale bar: 20 μm.

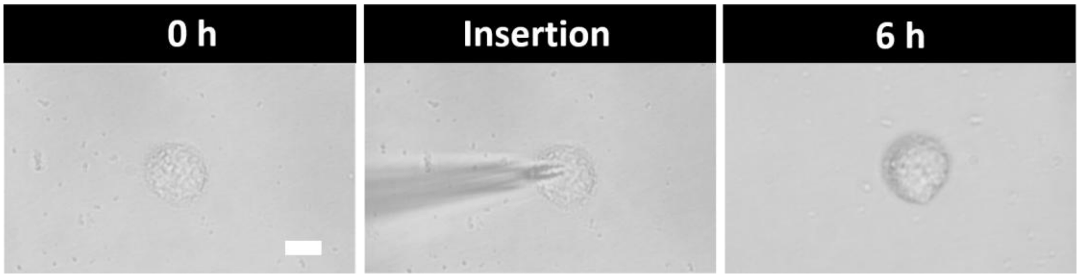

Figure S3. Microscopic images showing a MCF-7 cell before and after injection using a 100 nm nanopipette for different times (from left to right: BF images of a selected MCF-7 cell before injection, during injection, after injection in 6 h). Scale bar: 20 μm.

Supplementary Note 3. Experimental Platform for Single Cell Injection

The experimental setup used for single cell fluorescence imaging was illustrated in Fig. S4. To build the electrical connection, an Ag/AgCl electrode was immersed into the solution inside the nanopipette as the working electrode, and another Ag/AgCl electrode was dived into the culture medium as the reference electrode. Before inserting the cell, the nanopipette was fixed on a motorized micromanipulator under an inverted microscope for the injection of artificial monosaccharide into single cells. A single cell was chosen under microscope bright field imaging. By adjusting and controlling the nanopipette tip step by step using the electric driving microoperation system, the tip could be precisely controlled for approaching a single cell surface. Then, the tip was further moved downward until enter into the cytoplasm. Afterwards, a positive DC voltage was added to the nanopipette for the intracellular injection.

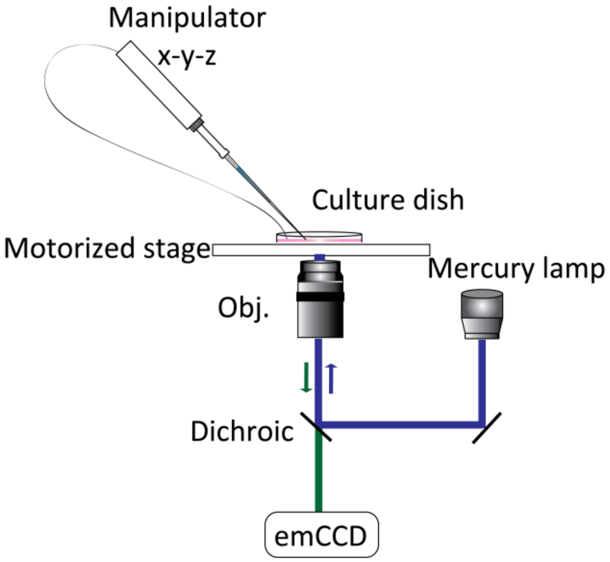

Figure S4. Schematic of the single cell injection experimental setup.

Supplementary Note 4. Simulations and Modelling of the Electroosmotic Flow

COMSOL Multiphysics 5.2a (COMSOL AB, Stockholm, Sweden) was used for the finite element simulations of the electroosmotic flow in the nanopipette. A 2D symmetric model was applied for the cone-shaped nanopipette using laminar flow and electrostatics module. The static electric field gradient at the tip of nanopipette was studied. The axial velocity profile of the electroosmotic flow was also calculated.

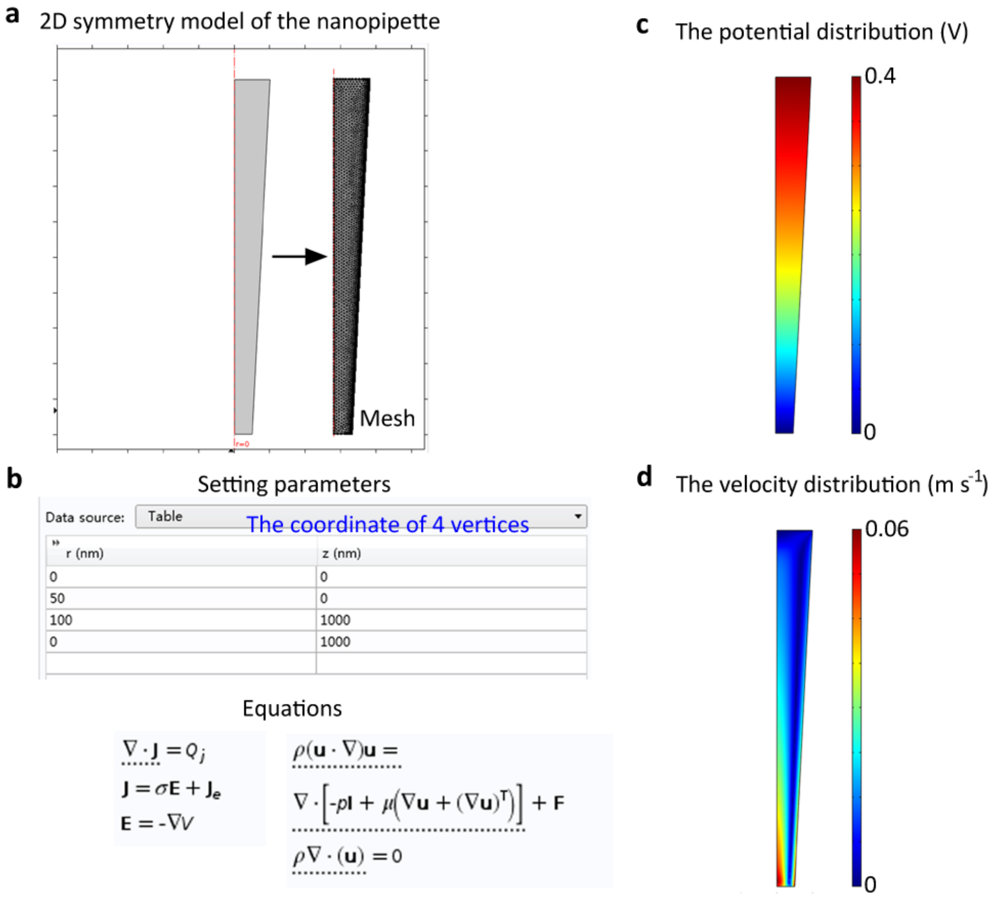

Figure S5. Simulations by COMSOL. (a) The 2D symmetric model of the nanopipette. (b) Parameters setting for the nanopipette and equations for calculation (laminar flow and electrostatics). (c) The potential distribution at the tip. (d) The velocity distribution of the electroosmotic flow at the tip.

Supplementary Note 5. Traditional Metabolic Labeling

The cells were seeded on confocal dishes and cultured for 12 h. Then the cells were marked by the following process: the cells were incubated with ManNAz (80 μM) in culture medium for 24 h. The cells were washed three times by the culture medium, and then incubated with AF 488- alkyne (15 μM) at 4 ºC for 30 min. After washing three times, the cells were observed under a microscope. The equipment parameters (including the laser power, gain, pinhole size, objective selection, acquisition channel, average, accumulate, etc.) were kept the same for all the cell imaging experiments. The fluorescence of AF 488 was obtained under 495 nm excitation.

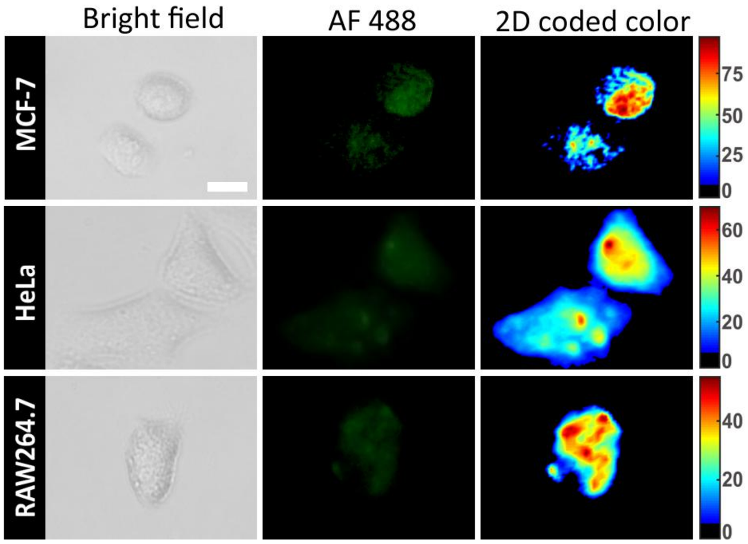

Figure S6. Microscopic images of MCF-7, HeLa and RAW264.7 cell treated with ManNAz for 24 h and then incubated with AF488 for 30 min. Scale bar: 20 μm. From left to right: bright field, green fluorescence, 2D color-coded green channel intensity.

Supplementary Note 6. Matlab Script for Acquiring 2D or 3D Coded Color Pseudogram of Fluorescence Cell Images

The color-coded pseudogram showing the green channel intensity of a cell image was acquired by Matlab r2017b. First, the cell image was opened, and the image matrix was opened by Matlab. Then the green channel was taken out. Next, the call surf function was used to draw the 3D distribution of cell fluorescence. To obtain 2D image, view (0,90) was added.

```
clc;close all
I = imread('filepath+filename');
J = imresize(I,[100 100]);%Resize the picture to 100*100 pixels
g = J(:,:,2);             %Take out green channel
k = jet;
colormap(k)
surf(g)
set(gca,'ydir','reverse');%Invert the y axis
shading interp
view(0,90)
colorbar
caxis([0 150])
```

Figure S7. Matlab script for acquiring pseudogram.

Supplementary Note 7. 3D Fluorescence Distribution of ManNAz Injected MCF-7 Cells

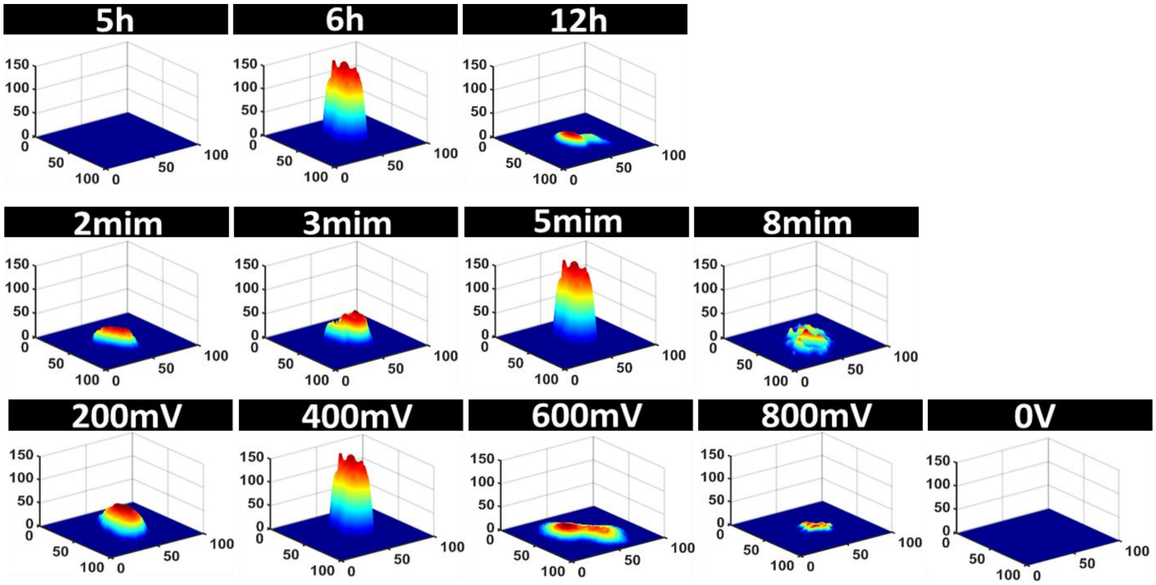

Figure S8. 3D color-coded pseudograms showing the green channel intensity of MCF-7 cells treated by different incubation time, injection time, and voltage (corresponding to Figure 2b-e in the manuscript).

Supplementary Note 8. Asymmetric Cell Division Observed in ManNAz Injected MCF-7 Cells

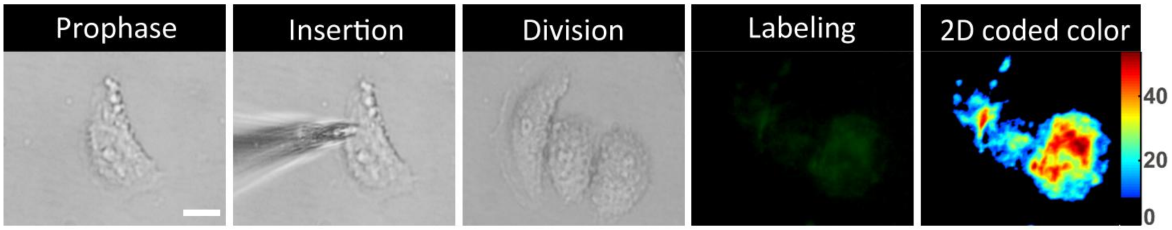

Figure S9. Microscopic images showing a dividing MCF-7 cell injected with ManNAz and incubated with AF488. From left to right: prophase, insertion, green fluorescence, 2D color-coded green channel intensity. Scale bar: 20 μm.

Supplementary Note 9. The Current Signal in the Injecting Progress

Fig. S10 showed the current signal diagram during the cell injection process under optimal conditions. When the nanopipette was inserted into the cell, a sudden change in the current could be observed. Afterwards, the current kept unchanged for the 5 min injection. Finally, the current dropped again as the nanopipette was moved out from the cell.

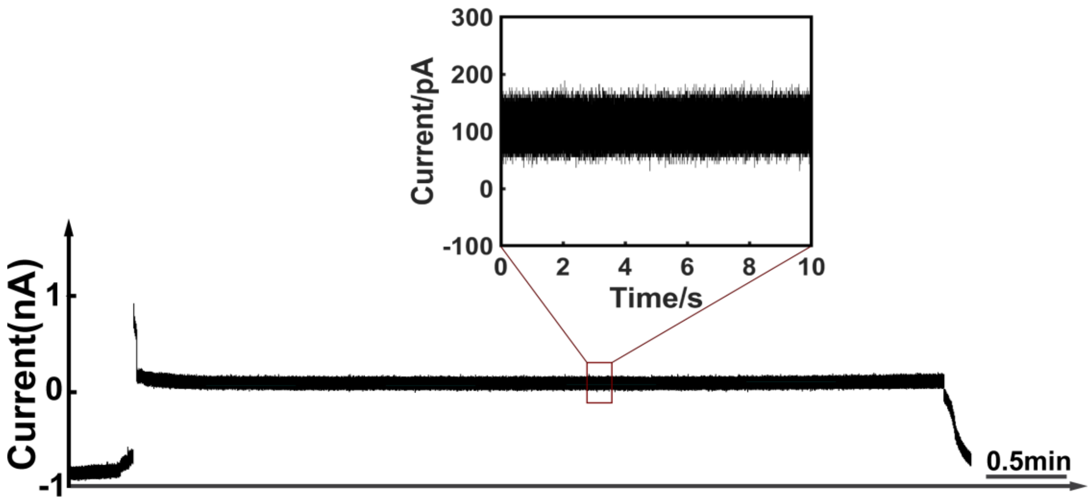

Figure S10. Current changes during the injecting process.

Supplementary Note 10. Control Experiments with Zero Potential

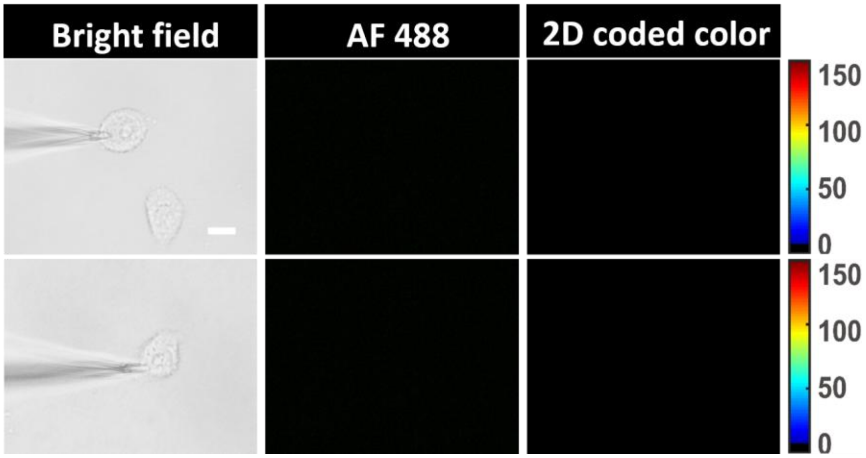

Figure S11. Microscopic images showing SiaNAz level under 0 mV injection voltage. From left to right: bright field, green fluorescence, 2D color-coded green channel intensity. Scale bar: 20 μm.

Supplementary Note 11. Division Process of MCF-7 Cells

We found that the asymmetric cell division occurred in MCF-7 cells treated by nanopipette injection. In order to test whether this phenomenon specifically happened to MCF-7 cells or not, the nanopipette injection experiments using different cells have been performed. As shown in Fig. S12, the asymmetric cell division occurred in the injected MCF-7 cell. Only MCF-7 cells were found to show the asymmetric division, and this phenomenon did not occur in HeLa or RAW264.7 cells.

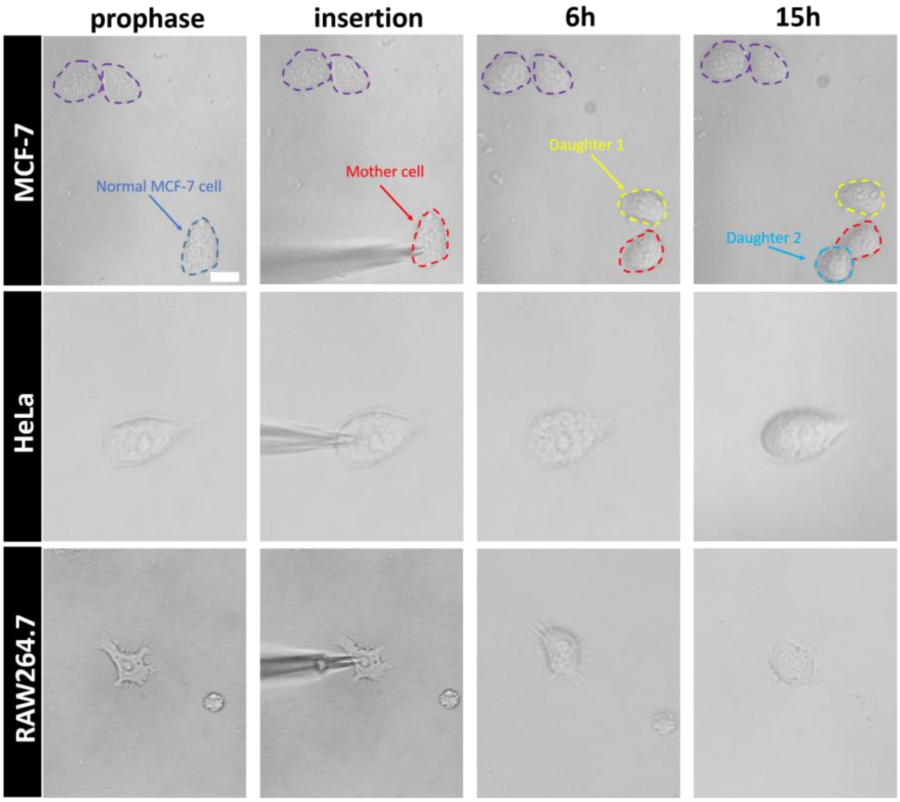

Figure S12. Microscopic observation of different cells before and after nanopipette injection. From left to right: prophase, insertion, 6 h after injection and 15 h after injection. Scale bar: 20 μm.

1. R. C. Qian, et al. *Anal. Chem.* **2018**, *90*, 13744-13750.

2. B. P. Nadappuram, et al. *Nat. Nanotechnol*. **2019**, *14*, 80-88.
